# Supplementary material for: The genome sequence of a cranefly, Tipula lateralis Meigen, 1804
Source: Wellcome Open Res. 2025 Mar 19;10:149. [Version 1] doi: 10.12688/wellcomeopenres.23899.1 (PMC12485334; doi:10.12688/wellcomeopenres.23899.1)
Supplement: Peer review history

# Reviewer report: Reviewer response for version 1

**DOI:** 10.21956/wellcomeopenres.26366.r121603

**Published:** 2025-05-02

**Authors:** Markus Friedrich

**Affiliations:** 1 Wayne State University, Michigan, USA

This report describes the chromosome-level sequencing of a female specimen of the cranefly species Tipula lateralis (Meigen, 1804) for the Darwin Tree of Life initiative. Data generation, processing, and curation are state of the art quality.

**Comments:**

 1. "Tipula intermedia shares the same BIN..." Define BIN

 2. Review inconsistent use of Tipula lateralis vs T. lateralis throughout major text body.

 3. Define INSDC

 4. Fig. 4: buscogenes = BUSCO genes

 5. The average transcript length is 8,869.40 ribonucleotides?

 6. "At a minimum, samples were required to have an average fragment size exceeding 8 kb and a total mass over 400 ng to proceed to the low input SMRTbell Prep Kit 3.0 protocol (Pacific Biosciences, California, USA), depending on genome size and sequencing depth required."

 Only one sample (abdomen of idTipLate1) was subjected to PacBio HiFi sequencing.

Are sufficient details of methods and materials provided to allow replication by others?

Yes

Is the rationale for creating the dataset(s) clearly described?

Yes

Are the datasets clearly presented in a useable and accessible format?

Yes

Are the protocols appropriate and is the work technically sound?

Yes

Reviewer Expertise:

Comparative genomics

I confirm that I have read this submission and believe that I have an appropriate level of expertise to confirm that it is of an acceptable scientific standard.

# Reviewer report: Reviewer response for version 1

**DOI:** 10.21956/wellcomeopenres.26366.r121600

**Published:** 2025-04-02

**Authors:** Marcus Stensmyr

**Affiliations:** 1 Lund University, Lund, Sweden

The submitted manuscript presents a high quality genome assembly of a cranefly (Tipula lateralis), sequenced as part of the Darwin Tree of Life project. The genome has been sequenced, assembled, and annotated using the state-of-the-art pipeline employed by the DToL project. Kudos to the authors for adding a nice and informative introduction, which these types of papers typically lack (at least the ones I have reviewed...). The provision of an additional crane fly genome assembly will aid efforts understanding the evolutionary history of this fun group of dipterans.

Are sufficient details of methods and materials provided to allow replication by others?

Yes

Is the rationale for creating the dataset(s) clearly described?

Yes

Are the datasets clearly presented in a useable and accessible format?

Yes

Are the protocols appropriate and is the work technically sound?

Yes

Reviewer Expertise:

Diptera neurogenetics and genomics

I confirm that I have read this submission and believe that I have an appropriate level of expertise to confirm that it is of an acceptable scientific standard.
